# Autoregulation of Acetylcholine Release and Micro-Pharmacodynamic Mechanisms at Neuromuscular Junction: Selective Acetylcholinesterase Inhibitors for Therapy of Myasthenic Syndromes

**DOI:** 10.3389/fphar.2018.00766

**Published:** 2018-07-12

**Authors:** Konstantin A. Petrov, Evgeny E. Nikolsky, Patrick Masson

**Affiliations:** ^1^A.E. Arbuzov Institute of Organic and Physical Chemistry, FRC Kazan Scientific Center of Russian Academy of Sciences, Kazan, Russia; ^2^Neuropharmacology Lab, Kazan Federal University, Kazan, Russia; ^3^Kazan Institute of Biochemistry and Biophysics, FRC Kazan Scientific Center of Russian Academy of Sciences, Kazan, Russia

**Keywords:** neuromuscular junction, myasthenia gravis, congenital myasthenic syndromes, cholinesterases, micro-pharmacodynamic mechanisms

## Abstract

Neuromuscular junctions (NMJs) are directly involved into such indispensable to life processes as respiration and locomotion. However, motor nerve forms only one synaptic contact at each muscle fiber. This unique configuration requires specific properties and constrains to be effective. The very high density of acetylcholine receptors (AChRs) of muscle type in synaptic cleft and an excess of acetylcholine (ACh) released under physiological conditions make this synapse extremely reliable. Nevertheless, under pathological conditions such as myasthenia gravis and congenital myasthenic syndromes, the safety factor can be markedly reduced. Drugs used for short-term symptomatic therapy of these pathological states, cause partial inhibition of cholinesterases (ChEs). These enzymes catalyze the hydrolysis of ACh, thus terminate its action on AChRs. Extension of the lifetime of ACh molecules compensates muscular AChRs abnormalities and, consequently, rescues muscle contractions. In this mini review, we will first outline the functional organization of the NMJ, and then, consider the concept of the safety factor and how it may be changed. This will be followed by a look at autoregulation of ACh release that influences the safety factor of NMJs. Finally, we will consider the morphological features of NMJs as a putative reserve to increase effectiveness of pathological muscle weakness therapy by ChEs inhibitors due to opportunity to use micro-pharmacodynamic mechanisms.

## Architecture and Physiology of NMJ

**FIGURE 1:**
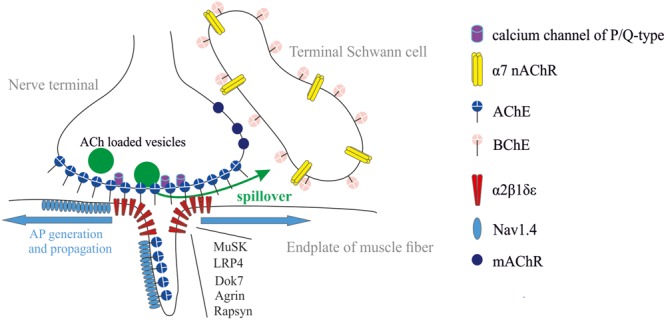
Tripartite organization of the NMJ: nerve terminal, muscle fiber, and terminal Schwann cell (TSC). Acetylcholinesterase (AChE) is mainly clustered in synaptic cleft. It controls the activation of muscle type AChR (α2βδ𝜀). Butyrylcholinesterase (BChE) is anchored at the TSC and controls activation of α7 nicotinic AChR (nAChR) and, probably, muscarinic (mAChRs). The most common forms of synaptic abnormalities are caused by autoantibodies to voltage-gated calcium channels of P/Q-type, α2β1δ𝜀, muscle-specific kinase (MuSK) and low-density lipoprotein-related protein 4 (LRP4). The most common congenital myasthenic syndromes are caused by mutations in genes coding for several functional proteins: (i) subunits of α2β1δ𝜀, (ii) proteins necessary for clustering of α2β1δ𝜀 (LRP4, Dok-7, agrin, rapsyn), and (iii) voltage-gated sodium channels (Nav1.4).

Neuromuscular junction (NMJ) is a synapse made up of motor axon branch (so-called nerve terminal), synaptic cleft, and postsynaptic region of muscle fiber (so-called end-plate), which is a folded structure where primary and secondary folds are distinguished. Such a configuration provides multiple extension of surface area allowing a huge density of receptors, ionic channels, and cholinesterases (ChEs) in a small crowded space (**Figure 1**). NMJ is a tripartite synapse because three to five terminal Schwann cells (TSCs) covering each nerve terminal actively participate in the process of neuromuscular synaptic transmission (Robitaille, 1995; Robitaille et al., 1997; Rochon et al., 2001; Todd et al., 2007, 2010; Ko and Robitaille, 2015; Arbour et al., 2017; Heredia et al., 2018).

The AP spreads along motor nerve fiber, leading to the temporal activation of voltage-gated calcium channels. The resulting acetylcholine (ACh) release as quanta, takes place at the active zone. ACh is loaded into presynaptic vesicles which fuse with presynaptic cell membrane and elbow out ACh into synaptic cleft (Slater, 2015, 2017; Jones et al., 2017; Badawi and Nishimune, 2018). It is worth noting that other neurotransmitters (glutamate, GABA, ATP) exist at NMJ where their putative role is fine tuning of ACh release (Silinsky and Redman, 1994; Correia-de-Sá et al., 1996; Petrov et al., 2013; Malomouzh et al., 2015; Nascimento et al., 2017).

Diffusing across synaptic cleft, ACh molecules reach postsynaptic membrane and bind to ACh receptors (AChRs) of muscle type (α2β1δ𝜀). AChRs occupancy, i.e., activation, results in the opening of coupled ionic channels. Inflow of sodium ions according to electrochemical gradient causes transient lowering of membrane potential in the postsynaptic region, i.e., generation of excitatory postsynaptic potential that are known as end-plate potential (EPP). In turn, EPP triggers the opening of voltage-gated sodium channels (Nav1.4) in muscle fiber and muscle AP generation. The time necessary for the whole system to recover determines the lability of synapse, i.e., the ability to reproduce the specific for each muscle pattern of excitation. This time depends on the rate of enzymatic ACh hydrolysis. During the falling phase of EPPs (2–3 ms), high rate of acetylcholinesterase (AChE)-catalyzed hydrolysis of ACh with a turnover approaching 1.5 × 10^4^ ACh molecules per second (Taylor et al., 2009) clears the synaptic cleft of all released ACh and, consequently, guarantees low probability of AChR activation. Thus, the main function of AChE in synaptic cleft is to ensure the rapid destruction of released ACh in the interstimuli intervals, before release of next quanta.

Mammalian ChEs family includes AChE (E.C. 3.1.1.7) and butyrylcholinesterase (BChE; E.C. 3.1.1.8) that are closely related enzymes. Their catalytic site is dedicated to hydrolyze ACh (Dvir et al., 2010; Masson and Lockridge, 2010). At NMJs, the most functional distinctions between these enzymes are associated with their localization. AChE at the NMJ is clustered mainly in synaptic cleft (Bernard et al., 2011; Blotnick-Rubin and Anglister, 2018), whereas BChE is mainly accumulated outside synaptic cleft, around TSCs (Davis and Koelle, 1967; Petrov et al., 2014) (**Figure 1**). Thus, ACh hydrolysis in synaptic cleft is accomplished by the sole AChE. AChE controls the lifetime of ACh and its occupancy time on AChRs in synaptic cleft. BChE, in turn, controls the spillover and dynamics of ACh outside synaptic cleft.

## Concept of Safety Factor and Pathological Muscle Weakness

AP is generated according to the principle “all or nothing.” It means that any depolarization of exceeding threshold results in generation of the same APs. The reliability of neuromuscular transmission results from the release of more ACh molecules than are required to depolarize the muscle fiber to the threshold of AP generation. Thus, the “safety factor” is expressed most often as the ratio of the estimated mean peak amplitude of EPPs to the threshold depolarization required to generate an AP in the muscle fiber (Wood and Slater, 2001; Slater, 2009).

At NMJ, there are two main points at which the safety factor of synaptic transmission can be compromised. These are reduction of ACh release and decreasing in efficiency of postsynaptic depolarization, caused by ACh (Ruff, 2011). Here we describe briefly some of the most common instances in which the safety factor of neuromuscular transmission is impaired.

The most common form of pathological muscle weakness is myasthenia gravis (MG). Weakness in MG is caused by autoantibodies directed against muscle type AChRs (80% of all MG patients), muscle-specific kinase (MuSK), and low-density lipoprotein-related protein 4 (LRP4) (**Figure 1**). Additional antigenic targets as agrin, collagen Q, titin, and ryanodine receptor have been described recently, but their pathogenicity and clinical significance are unclear so far. The cause of autoimmune response is unknown (Cavalcante et al., 2012; Howard, 2018; Kusner et al., 2018; Plomp, 2018). MG therapy approaches are ChEs inhibitors for short-term symptom control, immunosuppressive drugs for long-term modification of the disease course, and in some cases thymectomy. Treatments of acute exacerbations are plasmapheresis, immunoadsorption, and intravenous immunoglobulin (Verschuuren et al., 2016). A complement inhibitor, eculizumab was recently approved for the treatment of generalized MG. Other treatments including targeted monoclonal antibody agents are currently under investigation (Lee and Jander, 2017).

Lambert–Eaton myasthenic syndrome (LEMS) is the presynaptic disorder of neuromuscular transmission caused by decrease in the number of released ACh quanta. Weakness is caused by autoantibodies to voltage-gated calcium channels of P/Q-type (**Figure 1**). Around 60% of patients suffering from LEMS have an underlying malignancy, most commonly lung cancer. In other cases the trigger mechanism is unknown (Verschuuren et al., 2016). The most effective symptomatic treatment of LEMS involves administration of 3,4-diaminopyridine (Keogh et al., 2011). This drug blocks voltage-gated potassium channels in nerve terminals (**Figure 1**), thereby prolonging the AP. This enhances entry of Ca^2+^ into the nerve terminal and increases ACh release. Treatments proposed for long-term modification of this disease include immunosuppressants (Kesner et al., 2018).

The congenital myasthenic syndromes (CMS) are rare genetic disorders that are characterized by abnormal neuromuscular synaptic transmission. The most common forms of CMS are due to mutations in the genes coding for the different subunits of AChR. Other forms of CMS include mutations coding for different postsynaptic proteins (Dok-7, rapsyn, voltage-gated sodium channels), proteins present in the synaptic cleft (collagen Q, forming a tail that anchors AChE) (Legay, 2018), and presynaptic terminal proteins such as choline acetyltransferase (Ohno et al., 2001; Engel et al., 2015; Nicole et al., 2017) (**Figure 1**). For most of CMS syndromes that can be characterized physiologically as having “underactive” synapses, trial of a ChE inhibitor, and/or 3,4-diaminopyridine may be appropriate. The exception is for patients carrying a Dok-7 mutation who do not respond to ChE inhibitors. Some of CMS syndromes can be described physiologically as having “overactive” synapses (e.g., collagen Q deficiency and slow channel syndrome). In these cases, AChE inhibitors are contraindicated. Patients with slow channel syndrome may benefit from treatment of long-acting agents that block the AChR ion pore, such as quinidine or fluoxetine. Finally, albuterol or one of the β-agonists have been empirically found to benefit patients with Dok-7 and collagen Q deficiency (Engel, 2007; Engel et al., 2015).

Toxicant-induced NMJ pathologies include poisoning by inhibitors of ChEs and nicotinic AChR (nAChR) blockers (Pope et al., 2005; Pope and Brimijoin, 2018; Renew et al., 2018).

## Cholinesterase Inhibition and Autoregulation of Acetylcholine Release

At the moment, processes accompanying inhibition of ChEs at NMJ are very well studied at the level of single quantum release effects, by recording so-called miniature EPPs (mEPPs). These mEPPs are the result of release of a single ACh vesicle, which under resting conditions (absence of a nerve APs or in between two nerve APs) every few seconds fuse with the nerve terminal membrane so that small spontaneous EPPs can be recorded.

In this case, the sequences of inhibited synaptic AChE are: (i) more ACh molecules reach the postsynaptic membrane without being catalytically hydrolyzed by AChE during their diffusion across the synaptic cleft, and therefore, more AChRs are activated; (ii) because of prolonged lifespan in synaptic cleft, ACh molecules activate AChRs sustainably. Electrophysiological recordings show that prolonged lifetime of ACh in synaptic cleft due to AChE inactivation results in the increase of amplitude and duration of mEPPs (Katz and Miledi, 1975; Petrov et al., 2006, 2009, 2011). This fact is in agreement with idea that the main function of AChE in synaptic cleft is to control the duration of ACh action on postsynaptic AChRs.

Butyrylcholinesterase inhibition does not influence the mEPP amplitude and duration either when AChE is active or when this enzyme is inactivated (Minic et al., 2003). Thus, BChE inhibition does not potentiate the effect of ACh on muscle type AChRs. The absence of effects on mEPPs parameters due to BChE inhibition can be explained by localization of this enzyme outside the synaptic cleft on the surface of TSCs.

Since each EPP is the sum of the effect of individual mEPPs released simultaneously, it can be expected that the effects of AChE and BChE inhibition on mEPP and EPP are similar. However, electrophysiological recordings show that after complete inhibition of ChEs, EPPs amplitude varies, but in most cases, below the level of EPPs amplitude, recorded when ChEs are fully active. At the same time, mEPPs amplitude recorded in interstimuli intervals after ChEs inhibition is higher than mEPPs amplitude under conditions of active AChE and BChE. This suggests that when ChEs are inhibited, despite the amplification of postsynaptic effect of each quantum, fewer quanta of ACh are secreted in response to nerve AP. In other words, excess of ACh in synaptic cleft, resulting from ChEs inhibition, depresses ACh release.

Autoregulation of ACh release, i.e., the capability of ACh to modulate parameters of own secretion was described long time ago (Ciani and Edwards, 1963; Duncan and Publicover, 1979). It is known that presynaptic AChRs are of both types, ionotropic (nAChR) and metabotropic [muscarinic (mAChRs)]. It was shown that mAChRs, at least of four subtypes (Ì1, M2, M3, and Ì4), are present at NMJ (Garcia et al., 2005), although their physiological role is not fully established. It was shown that in some cases exogenous mAChRs agonists can suppress ACh release, while in other situations these agonists may enhance ACh release (Abbs and Joseph, 1981; Wessler et al., 1987; Arenson, 1991; Robitaille et al., 1997; Slutsky et al., 2001; Oliveira et al., 2002, 2009; Santafé et al., 2003, 2004, 2006, 2007; Dudel, 2007; Kovyazina et al., 2010, 2015).

It was shown that specific inhibition of AChE increases the probability of ACh release through activation of M1 mAChR subtype (Minic et al., 2002). On the contrary, it was shown that inhibition of both AChE and BChE decreases the probability of ACh release regardless the type of mAChRs (Minic et al., 2003).

The regulation of ACh release at NMJs by nAChRs is less documented compared to mAChRs. It was shown that α7 nAChRs can be localized at the TSC and act as a sensor for spillover of ACh adjusted by BChE (**Figure 1**). It was shown that ACh release was significantly depressed through the activation of α7 nAChR when BChE was specifically inhibited (Petrov et al., 2014). When both AChE in the synaptic cleft and BChE at TSC were inhibited, the spillover is increased. This induces a dramatic reduction of ACh release that compromises the muscle twitch triggered by the nerve stimulation.

It was shown that MG triggers homeostatic synaptic plasticity, resulting in increased ACh release. However, this pool of vesicles is small. Vesicles are rapidly depleted, leading to a larger depression in EPP amplitude during repetitive stimulations. This depression may contribute to the reduction of safety factor in patients with MG (Wang and Rich, 2018).

Under conditions of reduced safety factor, even slight downregulation in intensity of ACh release may influence muscle contraction. Because, the activation of AChRs, working as sensors at the input of different autoregulation pathways, is also controlled by AChE and BChE, the use of ChEs inhibitors does not only potentiate the effect of ACh on postsynaptic nAChRs, but also causes additional activation of presynaptic autoregulation pathways.

The use of selective AChE or non-selective (AChE and BChE) inhibitors to treat pathological muscle weakness was previously discussed (Komloova et al., 2010; Petrov et al., 2018a). In this review, we provide new evidences that selective AChE inhibitors could be even better than non-specific inhibitors to improve the muscle function. AChE inhibition increases the life-time of ACh molecules in the synaptic cleft and thus the number of nAChRs opened upon ACh binding. Indeed, AChE greatly contributes to make possible the jumping of EPP amplitude above the threshold of AP generation, and thus, the occurrence of muscle fibers twitch. With non-selective inhibitors, if BChE is also inhibited, the negative loop is stimulated. Then, less ACh quanta are released and EPP amplitude is reduced.

## Nmj and Opportunity to Use Micro-Pharmacodynamic Mechanisms

**FIGURE 2:**
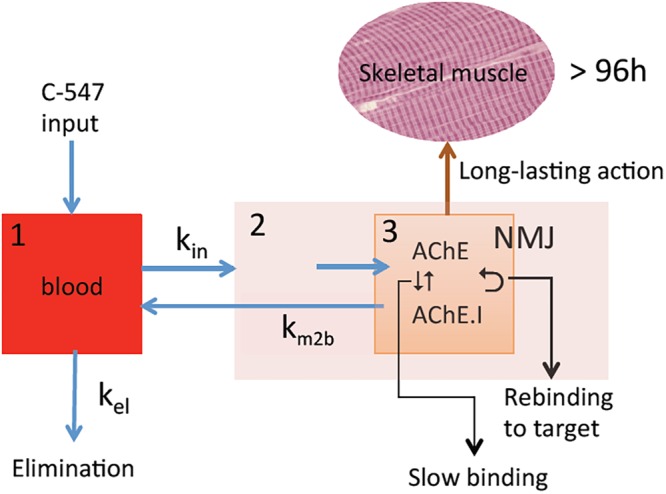
PK/PD model for the action of C-547 (I) at NMJ. **(1)** Central compartment (blood stream); **(2)** striatal muscle compartment; **(3)** NMJ sub-muscular compartment with high concentration of AChE (physiological target) in contained space. Slow-binding inhibition of AChE, long residence time of I on target, and slow elimination of I from NMJ lead to possibility of re-binding to AChE. This determines long-lasting action of C-547.

The NMJ architecture containing high density of AChE (5000 AChE monomers/μm^2^) (Anglister et al., 1994) and α2β1δ𝜀 nAChR (10,000 receptors/μm^2^) (Sine, 2002) in a small crowded space determines a sub-compartment at the origin of particular PK/PD mechanisms (**Figure 2**). The micro-anatomical structure of NMJ determines high target occupancy of ligands and increases residence time of these ligands on targets due possible rebinding. As a consequence, binding kinetics controls the duration of drug action in micro-anatomical compartments. Therefore, potent slow-binding ligands with slow rate of dissociation from targets display long-lasting action. The latter is important for the medical use AChE inhibitors, since pharmacological effect persists only while AChE at the NMJ is inhibited. In addition, faster elimination of inhibitors from myocardium and smooth muscles where the architecture of synapses is different from NMJ of skeletal muscles can reduce the duration of unwanted side effects.

Concepts and methodology for analysis of micro-PK/PD mechanisms and drug discovery have been developed in the past decade (see Copeland et al., 2006; Vauquelin, 2010, 2015, 2016; Vauquelin and Charlton, 2010; Copeland, 2011; Walkup et al., 2015; Tonge, 2018).

Very few slow-binding inhibitors of AChE have been used for their pharmacological properties and they have not been analyzed in terms of micro-PK/PD mechanisms. However, a recent PK/PD study of a potent and highly selective inhibitor of AChE, C-547 (Kharlamova et al., 2016), revealed micro-pharmacodynamic mechanisms taking place in NMJ (Petrov et al., 2018b).

**SCHEME 1:**
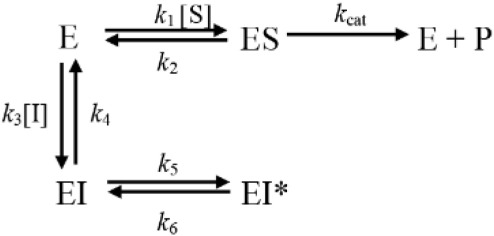
Mechanistic scheme for slow-binding inhibition of AChE by C-547.

C-547 is a slow-binding inhibitor of AChE of type B (for a review about slow-binding inhibitors, see Masson and Lushchekina, 2016). Slow-binding inhibitors of type B are ligands that bind rapidly to the enzyme, making a complex EI that slowly “isomerizes” to EI^∗^ (**Scheme 1**).

The isomerization step, in **Scheme 1**, corresponds to the crossing of the bottleneck in the active center gorge of the enzyme to reach its final position (Kharlamova et al., 2016; Masson and Lushchekina, 2016). The overall slow rate of dissociation of the enzyme inhibitor complex, *k*_off_, is (Eq. 1):

(1) $$\begin{array}{c} k_{\mathrm{off}}=\frac{k_{4}k_{6}}{\left(k_{4}+k_{5}+k_{6}\right)} \end{array}$$

The reciprocal of *k*_off_ is the residence time, τ, of ligand on AChE. The importance of this parameter has been emphasized as it determines the temporal duration of drug-target complex and leads to sustained pharmacology (Copeland, 2011).

Thus, in confined anatomical space such as NMJ where there is a high density of AChE subunits, binding kinetics of C-547 to AChE is characterized by long residence time on target (τ = 20 min) (Kharlamova et al., 2016) and slow diffusion rate of C-547 out of NMJ (Petrov et al., 2018b). This makes possible re-binding of C-547 to AChE, and therefore slow elimination from NMJ. In addition, binding of C-547 to albumin determines a slow distribution in tissues and long PK in the bloodstream (*t*_1/2_ = 3 h in rat). As the result of thermodynamic and kinetic selectivity, C-547 has a long-lasting action on skeletal muscles, higher than 72 h in rat model of MG. Compared to current drugs used for palliative treatment of MG, that display lower affinity and selectivity for AChE, short PK (*t*_1/2_ < 30 min) and short residence time (except for carbamates that form transient covalent adducts with AChE), binding kinetics and PK/PD of C-547 make this compound as a promising leader drug for improving sustained treatment of MG and related diseases. Therefore, the discovery of new drugs for treatment of NMJ diseases depends on identification of highly selective molecules that fast associate to targets (fast-on rates), show long residence times on target (slow-off rates) and display long PK in the bloodstream.

## Conclusion

Pharmacology of ChE inhibitors used for the treatment of muscle weakness is still poor. At the moment, only the carbamylating agent pyridostigmine bromide is used as ChEs inhibitor for treatment of muscle weakness symptoms. However, the selectivity of pyridostigmine for AChE versus BChE is low (6 against >10,000 for C-547; Petrov et al., 2018b). Taking into account ACh release down regulation under conditions of BChE inhibition, we can also conclude that selective inhibitors of AChE vs. BChE may have an advantage for MG treatment over non-selective ChE inhibitors, like pyridostigmine.

Moreover, symptomatic drug therapy often requires continuing sustained administration for maintaining high levels of target occupancy. Based on long-lasting target binding and rebinding, it is therefore possible to increase *in vivo* duration of drug action. Micro-anatomical properties of NMJ with high density of AChE could be helpful to design such long-lasting drugs that may greatly improve MG therapy. Design of new drugs, including selective AChE inhibitors, must be oriented in that direction.

## Author Contributions

KP wrote the manuscript in part associated with autoregulation of ACh release. PM wrote the manuscript in part associated with micro-pharmacodynamic mechanisms. EN suggested important information in order to improve the manuscript in part associated with NMJ architecture.

## Conflict of Interest Statement

The authors declare that the research was conducted in the absence of any commercial or financial relationships that could be construed as a potential conflict of interest.
